# Is a No-Restraint Policy Associated with Increased Aggression Towards Healthcare Professionals Among Inpatient Psychiatric Units? A 16-Year Retrospective Observational Study Conducted in Italy

**DOI:** 10.3390/nursrep14040276

**Published:** 2024-12-02

**Authors:** Marco Colizzi, Carla Comacchio, Marco Garzitto, Giovanni Napoli, Chiara Battiston, Tatiana Tam, Marco Bertoli, Calogero Anzallo, Alvisa Palese, Matteo Balestrieri

**Affiliations:** 1 Unit of Psychiatry and Eating Disorders, Department of Medicine (DMED), University of Udine, 33100 Udine, Italy; 2 Department of Psychosis Studies, Institute of Psychiatry, Psychology and Neuroscience, King’s College London, London SE5 8AF, UK; 3 Nursing and Midwifery Professional Service, Friuli Centrale Health University Authority, 33100 Udine, Italy; 4 General Hospital Psychiatric Unit (GHPU), Department of Addiction and Mental Health, Friuli Centrale Health University Authority, 33100 Udine, Italy; 5 Directorate, Department of Addiction and Mental Health, Friuli Centrale Health University Authority, 33100 Udine, Italy; 6 School of Nursing, Department of Medicine (DMED), University of Udine, 33100 Udine, Italy

**Keywords:** mental healthcare, healthcare professionals, workplace accidents

## Abstract

The aim of this study was to investigate aggression-related work accidents in an inpatient psychiatric unit before and after implementing a no-restraint policy in Italy. Results revealed that, over the study period (2007–2022), 113 accidents occurred, mostly related to physical aggression (81.4%), with healthcare assistants and psychiatric nurses being the most affected and more accidents occurring during the morning shift (49.6%). A transitory peak of accidents occurred during the policy transition (χ^2^_2_ = 16.0, *p* < 0.001; V = 1.000), falling rapidly in the subsequent years. In conclusion, adopting a no-restraint policy is not associated with increased aggression toward staff in psychiatric healthcare in the longer term, although greater support is needed during the transition phase to minimize risks.

## 1. Introduction

Workplace violence represents a major concern in healthcare settings, with nurses and physicians being the most exposed occupational categories and psychiatric and emergency departments being the services at greatest risk of violence [1]. In terms of patients’ characteristics, compulsory hospital admission, substance abuse, and lifetime history of violence have been associated with aggressive behavior towards healthcare professionals in mental health settings [2,3]. Of interest, mental health services perceiving a heightened risk of violence have been reported to be more likely to implement coercive measures such as seclusion, restraint, and enforced medication, the latter being perceived as traumatic by patients. Such procedures have been associated with aggressive responses instead of engagement and treatment compliance, leading to a vicious cycle [2].

The need to adopt a no-restraint policy in mental health inpatient units is widely debated [4] but rarely implemented, with recent position statements calling for the implementation of alternatives to coercion in psychiatric settings [5]. In fact, only 6% of general hospital psychiatric units (GHPU) across Italy are no-restraint, as found in a recent census (https://www.vita.it/psichiatria-ecco-i-19-reparti-dove-non-si-lega-nessuno, (accessed on 14 August 2024)). Also, the pros and cons of such a policy, especially in terms of risk of aggression and violence, have been poorly investigated [6]. In Italy, GHPUs are located within hospital facilities, are provided with common spaces, and have no more than 16 beds. In a ‘No restraint’ GHPU, no pharmacological or physical restraints are adopted, including bed restraints, and the doors of the ward are kept open throughout the day. In our GHPU, the transition to a no-restraint policy involved the following: (a) staff debriefing of aggression and violence episodes; (b) additional care of ward facilities; (c) early psychosocial interventions; (d) implementation of crisis-resolution protocols; (e) staff training on de-escalation techniques.

The aim of this study was to investigate the occurrence and characteristics of accidents at work related to aggression and violence in an Italian GHPU and how this has changed since the implementation of an open-door, no-restraint policy. In the ongoing debate on the adoption of no-restraint policies in mental health, we focused on the confounding effect that might be related to the transition between different policies.

## 2. Materials and Methods

A retrospective observational study collected data on workplace accidents occurring to healthcare professionals at the GHPU of an academic hospital located in the north of Italy and serving around 500,000 citizens for those health emergencies requiring admission. Data collection authorization was obtained from the hospital. The observation period was from 2007 to 2022, with 2015 being the year when the open-door, no-restraint policy was fully implemented. Data included the following: (i) accident type (physical aggression; accidental trauma; biological risk); (ii) accident prognosis (significant prognosis, >0 days; duration of prognosis, days); (iii) pre/post no-restraint policy accident occurrence; (iv) healthcare professionals’ (a) gender (female; male), (b) age (mean; by decade; 4-level), (c) schooling (professional; high school; degree 3-years; degree; more than degree) and (d) professional role (registered nurse, educated at university level as almost all nurses currently employed in the healthcare system; psychiatric nurse, not educated at university level but with a training course, a role established with the asylum and progressively discontinued; healthcare assistant, a supporting role who has undergone a training course; ward doctor, a medical doctor with a postgraduate formation in psychiatry); and (v) shift schedule’s characteristics (morning; afternoon/night). Additional information included the following: (vi) personnel mean-presence at the GHPU; (vii) personnel per bed (ratio); and (viii) personnel worked-time (months). All workplace accidents reported by personnel were included, as recorded in administrative records. According to the study design, the Strengthening the Reporting of Observational Studies in Epidemiology (STROBE) guidelines [7] were adopted to report methods and findings (see Supplementary Table S1).

### 2.1. Statistics

Absolute and/or percentage frequencies, means with standard deviations (SD), and range of variation were used to describe categorical and continuous variables, respectively. Normality was assessed with the Shapiro–Wilk test. In the preliminary and univariate analyses, the χ^2^ test (reporting values and degrees of freedom, df) or Fisher exact test (also reporting the odds ratios, OR, and confidence interval, ci, at 95% of the corresponding cross-tabulation) were used to evaluate data distribution. The effect sizes were evaluated using Cramer V for the χ^2^ test (small: row-wise minimum value equal to 2: 0.10 ≤ V < 0.30; equal to 3: 0.07 ≤ V < 0.21; medium: 2: 0.30 ≤ V < 0.50; 3: 0.21 ≤ V < 0.35; or large: 2: V ≥ 0.50; 3: V ≥ 0.35).

In comparisons between groups for continuous variables, homoscedasticity was verified by using Levene’s test centered on the median. Based on homoscedasticity, either parametric (*t*-test for independent samples (t, df); one-way analysis of variance between groups (F, df of the numerator and denominator)) or non-parametric tests (Mann–Whitney rank test (U); Kruskal–Wallis test (K)) were used in the comparisons. The effect sizes were evaluated using ω^2^ for one-way analyses of variance between groups (small: 0.01 ≤ ω^2^ < 0.06; medium: 0.06 ≤ ω^2^ < 0.14; large: ω^2^ ≥ 0.14). Univariate association between continuous measures was assessed using Spearman rank correlations (ρ). When evaluating effect sizes, correlations were considered negligible if less than or equal to ±0.100, weak if less than ±0.300, moderate if less than ±0.700, and strong if greater than ±0.700. As an observation unit, personnel comparisons were made on participants, accident comparisons mainly on specified time intervals.

The trend figures with bands indicating the 95% CI were obtained with a fit calculated with local polynomial regression. Statistical significance was conventionally set at α = 0.050 without corrections for multiple independent comparisons. The Tukey method (Honest Significant Differences) was adopted for post-hoc analyses of the analyses of variance. All analyses were carried out with R version 4.3.1 (of the R Foundation for Statistical Computing).

### 2.2. Ethics

This study was conducted in accordance with the Declaration of Helsinki. It was part of a larger project approved by the local Institutional Review Board (45/2023) at the Department of Medicine (DMED) of the University of Udine (a public institution obligated by the European Union’s General Data Protection Regulation). The anonymity of the information collected was guaranteed for the entire duration of the study, avoiding the acquisition of identifying information of individual persons (e.g., name, address, email address, etc.), and researchers involved in the study could not link accident data with healthcare professionals’ identities.

## 3. Results

Over the study period (2007–2022), there were 101 healthcare professionals working at the GHPU. Their sociodemographic and professional characteristics are reported in Supplementary Table S2. Over the years, an increase in the number of ward doctors was observed, resulting in an increased ratio per bed (ρ = +0.836, *p* < 0.001). Also, staff composition changed over time, with the retirement of psychiatric nurses from the workforce starting in 2016. As mentioned above, the transition to a no-restraint policy required a complex readjustment of the GHPU, also involving new routines.

GHPU characteristics in terms of accidents, personnel, and change of policy (open-door, no-restraint) are reported for the entire period, by year, in Supplementary Table S3. Significant variability was observed across years for accidents (range: 2–16) and physical aggressions (range: 1–14).

A total of 113 work accidents, with an average of 7.06 accidents per year, were recorded. Supplementary Table S4 provides a general description of the phenomenon. Most accidents were related to physical aggression (81.42%), with a significant correlation with the number of total accidents (ρ = +0.913, *p* < 0.001; Supplementary Figure S1). The average duration of prognosis was 5.75 ± 13.758 days.

Accidents were significantly associated with the professional role (χ^2^_3_ = 8.36, *p* = 0.039; V = 0.288), with a higher proportion among healthcare assistants and psychiatric nurses than registered nurses and ward doctors (Supplementary Figure S2). Without correction for multiple independent comparisons, ward doctors had fewer accidents than psychiatric nurses (*p* = 0.038; OR = 0.082 [<0.01, 1.20]) and healthcare assistants (*p* = 0.024; OR = 0.189 [0.03, 0.92]); other direct comparisons were not statistically significant (all with *p* ≥ 0.167). It should be noted that psychiatric nurses did not experience any accidents during the transition year (2015) as well as in the final period before their retirement from service (2016). Also, more accidents occurred during the morning shift (49.56%) than during the afternoon and night shifts. Instead, having had accidents was not found to be significantly associated with gender (*p* > 0.999; OR = 0.938 [0.397, 2.209]), birth-year (χ^2^_2_ = 2.12, *p* = 0.346), or schooling (χ^2^_2_ = 3.90, *p* = 0.142) of the healthcare professional. Similarly, socio-demographic characteristics were not found to be significantly associated with having had multiple accidents, type, and accident characteristics (i.e., prognosis, presence of physical aggression). We also did not observe statistically significant correlations between the number of accidents (or accidents due to physical aggression) and the service characteristics in terms of personnel presence and work intensity (all with *p* ≥ 0.140, with −0.220 ≤ ρ ≤ +0.386). Neither the increase in the number of ward doctors (ρ = −0.217, *p* = 0.420) nor other characteristics of the accidents were found to be significantly associated with the number of accidents.

**Figure 1:**
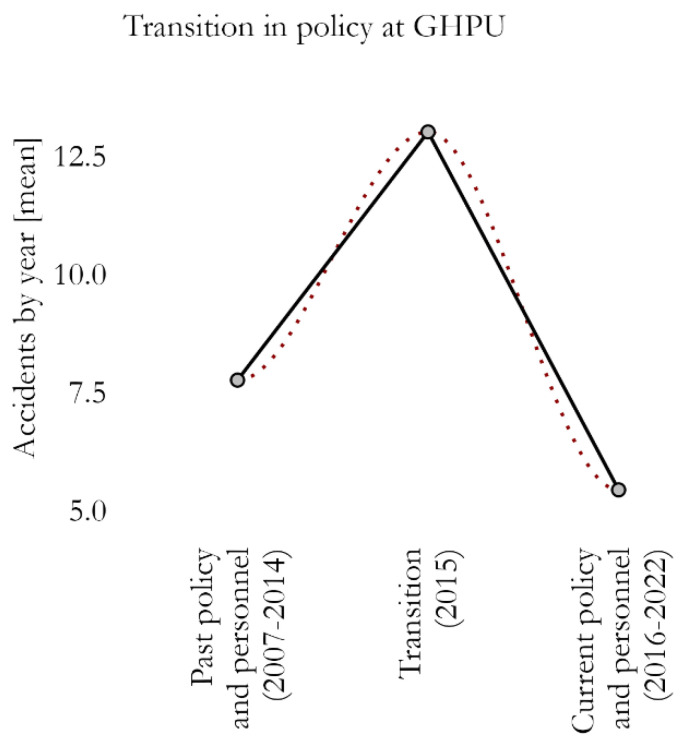
Average number of accidents per year according to the transition in the organizational model of the General Hospital Psychiatric Unit (GHPU). The fitting obtained with a local polynomial regression shows smoothed mean frequency (red dotted line).

When dividing the observations into three periods (over 10 years ago; 6–10 years ago; last 5 years), there was a significant difference in the distribution of accidents (F_2,13_ = 6.33, *p* = 0.012; ω^2^ = 0.400 [<0.01, +0.66]), with fewer in the last five-year period as compared to the intermediate one (*p* = 0.010; Supplementary Figure S3). Consistently, a transitory peak of accidents was observed during the policy transition, with the year 2015 being significantly associated with a rise in the frequency of accidents (χ^2^_2_ = 16.00, *p* < 0.001; V = 1.000; Figure 1; for details, see also Supplementary Figure S4), that fell rapidly in the subsequent years.

## 4. Discussion

This study investigated the effects of adopting a no-restraint policy in a GHPU in terms of workplace accidents due to physical aggression. During the 16-year observation period (2007–2022), around seven accidents/year occurred, mostly due to physical assaults, confirming previous evidence [8]. In fact, previous studies suggest that physical aggression toward healthcare professionals is a remarkable phenomenon, especially in psychiatric settings [9]. With respect to professionals involved, healthcare assistants and psychiatric nurses were more affected than registered nurses (educated at the university level) and doctors, likely because both trained at the vocational level and were less equipped with complex relational competence, in addition to their different work experience, professional role [10], and reported high levels of dissatisfaction with work [8], which can result in an increased risk for violence. Previous evidence from the Italian context showed that 74% of nurses may have suffered at least one episode of violence in their last 3 years of work, increasing to 84% when considering nurses working in mental health settings [9]. Our results showed a lower accident rate considering all nurses but a similar rate considering psychiatric nurses only.

In line with previous reports [8,10], a substantial proportion of aggressions occurred during the morning shift (49.56%), likely because patients are more stressed due to therapeutic program communications, and staff is less available, as involved in a greater number of activities.

Of interest, an increase in aggression-related accidents was observed around the year when the organizational model changed to an open-door, no-restraint policy. This finding may reflect the initial phase of adjustment of both staff and patients to the new policy, underlining the importance of carefully managing such a transition phase. In fact, studies highlight the crucial role of organizational protocols and training (e.g., procedure for self- and hetero-directed aggressive risk management) to support the full application of a no-restraint policy [11]. Also, it has been found that ward staff need to look for innovative solutions and more strenuously engage in the therapeutic relationship with the patient [12]. Regardless, the increase in work accidents due to physical aggression was transitory and self-limiting, returning to the levels observed before the policy implementation in the longer term.

Among the strengths of this study are the long temporal observation and the availability of data not only in terms of the number of accidents but also of time-shift of accidents and involved healthcare professionals. Also, changes occurring at the service level were continuously documented across the observation period (e.g., personnel and policy changes). The main limitation of this study is the collection of data from a single service, in the absence of comparability data from a GHPU that continued to adopt a restraint policy. In addition, collecting data from a single service resulted in a limited number of observations (especially when considering accidents by personnel characteristics), thus requiring confirmation in larger samples, and this prevented the opportunity to reliably analyze specific groups. Further, the causes and management of patient aggression and violence were not investigated, urging local needs and practices to be part of the entire evaluation process [13]. Considering the limitations presented above and the possibility of spurious associations between observed events, it is not possible to hypothesize a causal inference for the increase in aggression-related accidents during the transition to the new policy. Future studies will have to focus on external benchmarking of no-restraint policies for a direct comparison of health system performances at a wider level and to systematically analyze the contribution of each action related to the policy change.

## 5. Conclusions

In conclusion, the study suggests that the adoption of a no-restraint policy is not associated with an increase in the incidence of aggression in psychiatric healthcare in the longer term. However, concerns arise regarding the transition period, when a transitory increase in aggression-related work accidents may be expected, especially in nurses and healthcare assistants, as well as during specific time shifts. Thus, findings advocate offering greater organizational support and training to inpatient psychiatric services while transitioning to a no-restraint policy to minimize the risk of aggression to healthcare professionals.

## Data Availability

Data available on request due to restrictions, e.g., privacy or ethical.

## Supplementary Materials

The following supporting information can be downloaded at https://www.mdpi.com/article/10.3390/nursrep14040276/s1; Table S1: Checklist of the Strengthening the Reporting of Observational Studies in Epidemiology (STROBE); Table S2: Healthcare professionals’ characteristics (N = 101); Table S3: General Hospital Psychiatric Unit characteristics over the study period (from 2007 to 2022). A total of 113 work accidents were recorded; Table S4: Accidents’ characteristics over the study period (from 2007 to 2022). A total of 113 work accidents were recorded in total personnel of 101 healthcare professionals; Figure S1: Association between total accidents and accidents due to physical aggression over the study period (from 2007 to 2022). A total of 113 work accidents were recorded in total personnel of 101 healthcare professionals; Figure S2: Association between accident and professional role in healthcare professionals in the General Hospital Psychiatric Unit over the study period (from 2007 to 2022). A total of 113 work accidents were recorded in total personnel of 101 healthcare professionals; Figure S3: Frequency of accidents (N = 113) per year, in total personnel of 101 healthcare professionals; Figure S4: Average number of accidents (N = 113) per year according to the transition in the organizational model of the General Hospital Psychiatric Unit (GHPU) over the study period (from 2007 to 2022). Observations are organized by professional role in total personnel of 101 healthcare professionals (52 registered nurses, 63 accidents; 5 psychiatric nurses, 7 accidents; 30 healthcare assistants, 38 accidents; 14 ward doctors, 5 accidents).

## Public Involvement Statement

No public involvement in any aspect of this research.

## References

[1] Magnavita N., Heponiemi T. (2012). Violence towards health care workers in a Public Health Care Facility in Italy: A repeated cross-sectional study. BMC Health Serv. Res..

[2] Iozzino L., Ferrari C., Large M., Nielssen O., de Girolamo G. (2015). Prevalence and Risk Factors of Violence by Psychiatric Acute Inpatients: A Systematic Review and Meta-Analysis. PLoS ONE.

[3] d’Ettorre G., Pellicani V. (2017). Workplace Violence Toward Mental Healthcare Workers Employed in Psychiatric Wards. Saf. Health Work.

[4] Newton-Howes G., Savage M., Arnold R., Hasegawa T., Staggs V., Kisely S. (2020). The use of mechanical restraint in Pacific Rim countries: An international epidemiological study. Epidemiol. Psychiatr. Sci..

[5] Gill N., Drew N., Rodrigues M., Muhsen H., Morales Cano G., Savage M., Pathare S., Allan J., Galderisi S., Javed A. (2024). Bringing together the World Health Organization’s QualityRights initiative and the World Psychiatric Association’s programme on implementing alternatives to coercion in mental healthcare: A common goal for action. BJPsych Open.

[6] Schneeberger A., Kowalinski E., Fröhlich D., Schröder K., von Felten S., Zinkler M., Seine K., Heinz A., Borgwardt S., Lang U. (2017). Aggression and violence in psychiatric hospitals with and without open door policies: A 15-year naturalistic observational study. J. Psychiatr. Res..

[7] von Elm E., Altman D.G., Egger M., Pocock S.J., Gøtzsche P.C., Vandenbroucke J.P., Initiative S. (2007). The Strengthening the Reporting of Observational Studies in Epidemiology (STROBE) statement: Guidelines for reporting observational studies. Lancet.

[8] Franchini L., Colombo C., Aiolfi I., Alajmo V., Beckman E., Marcocci L., Ragone N., Travaini G. (2020). A descriptive study of suffered and witnessed aggressions in two rehabilitative italian units. Clin. Neuropsychiatry.

[9] Ferri P., Reggiani F., Di Lorenzo R. (2011). Aggressive behavior toward nursing staff in three different health care settings. Prof. Inferm..

[10] Bizzarri J., Piacentino D., Kotzalidis G., Moser S., Cappelletti S., Weissenberger G., Pompili M., Conca A. (2020). Aggression and Violence Toward Healthcare Workers in a Psychiatric Service in Italy A Retrospective Questionnaire-Based Survey. J. Nerv. Ment. Dis..

[11] Pocobello R., Camilli F., Rossi G., Davì M., Corbascio C., Tancredi D., Oretti A., Bonavigo T., Galeazzi G.M., Wegenberger O. (2024). No-Restraint Committed General Hospital Psychiatric Units (SPDCs) in Italy-A Descriptive Organizational Study. Healthcare.

[12] Di Napoli W., Andreatta O. (2014). A “no-restraint” psychiatric department: Operative protocols and outcome data from the “Opened-doors experience” in Trento. Psychiatr. Danub..

[13] Duxbury J., Whittington R. (2005). Causes and management of patient aggression and violence: Staff and patient perspectives. J. Adv. Nurs..

