# Supplementary material for: Is a No-Restraint Policy Associated with Increased Aggression Towards Healthcare Professionals Among Inpatient Psychiatric Units? A 16-Year Retrospective Observational Study Conducted in Italy
Source: Nurs Rep. 2024 Dec 2;14(4):3779–85. doi: 10.3390/nursrep14040276 (PMC11677375; doi:10.3390/nursrep14040276)
Supplement: Supplementary file 1 [file nursrep-14-00276-s001.zip › nursrep-3182836-supplementary.pdf]

Supplementary Table S1. Checklist of the Strengthening the Reporting of Observational Studies in Epidemiology (STROBE).

Item	No	Recommendation	Notes	
**Title and abstract**	1	(*a*) Indicate the study's design with a commonly used term in the title or the abstract.	The study is described as retrospective observational (see in the **Title**).	✓
		(*b*) Provide in the abstract an informative and balanced summary of what was done and what was found.	Study period, data collection, and main results are reported (see in the **Abstract**).	✓
**Introduction**				
Background/rationale	2	Explain the scientific background and rationale for the investigation being reported.	Background, research context and current debate are introduced (see in the first two paragraphs of the **Introduction** section).	✓
Objectives	3	State specific objectives, including any prespecified hypotheses.	Study aim and hypothesis are indicated (see last paragraph of the **Introduction** section).	✓
**Methods**				
Study design	4	Present key elements of study design early in the paper.	The study design is clearly described (see first paragraph of the **Materials and Methods** section).	✓
Setting	5	Describe the setting, locations, and relevant dates, including periods of recruitment, exposure, follow-up, and data collection.	Study period and data collection are indicated (see first paragraph of the **Materials and Methods** section).	✓
Participants	6	(*a*) Give the eligibility criteria, and the sources and methods of selection of participants. Describe methods of follow-up.	All available information is included, the study design does not include follow-ups (see first paragraph of the **Materials and Methods** section).	✓
		(*b*) For matched studies, give matching criteria and number of exposed and unexposed.	The study design does not include matching (see first paragraph of the **Materials and Methods** section).	✓
Variables	7	Clearly define all outcomes, exposures, predictors, potential confounders, and effect modifiers. Give diagnostic criteria, if applicable.	All collected measures are listed (see first paragraph of the **Materials and Methods** section).	✓
Data sources/ measurement	8	For each variable of interest, give sources of data and details of methods of assessment (measurement). Describe comparability of assessment methods if there is more than one group.	Source of information for data collection is indicated, the study design does not include subgroups (see first paragraph of the **Materials and Methods** section).	✓
Bias	9	Describe any efforts to address potential sources of bias.	All available information is included without selection (see first paragraph of the **Materials and Methods** section). Study limitations are intrinsic to the study design and discussed (see fourth paragraph of the **Discussion** section).	✓
Study size	10	Explain how the study size was arrived at.	All available information is included (see first paragraph of the **Materials and Methods** section).	✓

Item	No	Recommendation	Notes	
Quantitative variables	11	Explain how quantitative variables were handled in the analyses. If applicable, describe which groupings were chosen and why.	All collected measures are listed (see first paragraph of the **Materials and Methods** section). The handling of variables is described (see the **Statistics** sub-section, in the **Materials and Methods** section). Categorisation of measures is detailed when applicable (see first paragraph of the **Materials and Methods** section; see last paragraph of the **Results** section).	✓
Statistical methods	12	(*a*) Describe all statistical methods, including those used to control for confounding.	Statistical methods are detailed (see the **Statistics** sub-section, in the **Materials and Methods** section).	✓
		(*b*) Describe any methods used to examine subgroups and interactions.	The study design does not include subgroups (see first paragraph of the **Materials and Methods** section). Management of by-category comparison are described (see the **Statistics** sub-section, in the **Materials and Methods** section).	✓
		(*c*) Explain how missing data were addressed.	All available information is included (see first paragraph of the **Materials and Methods** section). There are no missing data.	✓
		(*d*) If applicable, explain how loss to follow-up was addressed.	The study design does not include follow-ups (see first paragraph of the **Materials and Methods** section).	✓
		(*e*) Describe any sensitivity analyses.	Statistical methods do not include sensitivity analyses (see the **Statistics** sub-section, in the **Materials and Methods** section).	✓
**Results**				
Participants	13	(a) Report numbers of individuals at each stage of study—e.g., numbers potentially eligible, examined for eligibility, confirmed eligible, included in the study, completing follow-up, and analysed.	Numbers of observations are reported (see **Results** section and **Supplementary Materials**). All available information is included, the study design does not include follow-ups (see first paragraph of the **Materials and Methods** section). There are no missing data.	✓
		(b) Give reasons for non-participation at each stage.	All available information is included (see first paragraph of the **Materials and Methods** section). There are no missing data.	✓
		(c) Consider use of a flow diagram.	All available information is included (see first paragraph of the **Materials and Methods** section). There are no missing data.	✓
Descriptive data	14	(a) Give characteristics of study participants (e.g., demographic, clinical, social) and information on exposures and potential confounders.	Details on study participants are reported (see first paragraph of the **Results** section and **Supplementary Materials**). The study design is simple (see first paragraph of the **Materials and Methods** section).	✓

Item	No	Recommendation	Notes	
		(b) Indicate number of participants with missing data for each variable of interest.	All available information is included (see first paragraph of the **Materials and Methods** section). There are no missing data.	✓
		(c) Summarise follow-up time (e.g., average and total amount).	The study design does not include follow-ups (see first paragraph of the **Materials and Methods** section).	✓
Outcome data	15	Report numbers of outcome events or summary measures over time.	Numbers of outcome events are reported also in relation to year of observation (see all paragraphs of the **Results** section and **Supplementary Materials**).	✓
Main results	16	(*a*) Give unadjusted estimates and, if applicable, confounder-adjusted estimates and their precision (e.g., 95% confidence interval). Make clear which confounders were adjusted for and why they were included.	Estimates with their 95% confidence intervals are reported when applicable (see fourth paragraph of the **Results**). Confounders are only used to obtain percentages (see all paragraphs of the **Results** section and **Supplementary Materials**).	✓
		(*b*) Report category boundaries when continuous variables were categorized.	Category boundaries are reported (see first paragraph of the **Materials and Methods** section; see last paragraph of the **Results** section).	✓
		(*c*) If relevant, consider translating estimates of relative risk into absolute risk for a meaningful time period.	The study design is simple and does not allow to formally translate estimates from relative risk to absolute risk for a meaningful time period (see first paragraph of the **Materials and Methods** section). Proportions were used to promote understanding of the relationships among events, time, and categories (see all paragraphs of the **Results** section and **Supplementary Materials**).	✓
Other analyses	17	Report other analyses done—e.g., analyses of subgroups and interactions, and sensitivity analyses.	The results of all statistically significant analyses are reported when applicable (see all paragraphs of the **Results** section and **Supplementary Materials**).	✓
**Discussion**				
Key results	18	Summarise key results with reference to study objectives.	Aim of the study and main results are reiterated to summarize them (see first and second paragraphs of the **Discussion** section).	✓
Limitations	19	Discuss limitations of the study, taking into account sources of potential bias or imprecision. Discuss both direction and magnitude of any potential bias.	Study limitations are discussed, it is pointed out that these limitations do not allow for specific in-depth analyses, generalizations, and confident causal inferences (see fourth paragraph of the **Discussion** section).	✓
Interpretation	20	Give a cautious overall interpretation of results considering objectives, limitations, multiplicity of analyses, results from similar studies, and other relevant evidence.	A conclusive paragraph tries to summarise the study results (see last paragraph of the **Discussion** section).	✓

Item	No	Recommendation	Notes	
Generalisability	21	Discuss the generalisability (external validity) of the study results.	It is pointed out that study limitations do not allow generalizations, without further studies (see fourth paragraph of the **Discussion** section).	✓
**Other information**				
Funding	22	Give the source of funding and the role of the funders for the present study and, if applicable, for the original study on which the present article is based.	This research received no external funding (see the **Funding** statement).	✓

Adapted from: https://www.strobe-statement.org/checklists. **Note:** Information on the STROBE Initiative is available at http://www.strobe-statement.org.

Supplementary Table S2. Healthcare professionals' characteristics (N=101).

**Measure**	**N (%) by-category** or **Mean ±SD [min, Max]**
Gender	Female: 55 (54.46%)
	Male: 46 (45.54%)
Age-group (by decade)	′50s: 15 (14.85%)
	′60s: 31 (30.69%)
	′70s: 24 (23.76%)
	′80s: 24 (23.76%)
	′90s: 7 (6.93%)
Schooling	Professional: 30 (29.70%)
	High school: 25 (24.75%)
	Degree 3-years: 26 (25.74%)
	Degree: 16 (15.84%)
	More than degree: 4 (3.96%)
Professional role	Registered nurse: 52 (51.49%)
	Psychiatric nurse: 5 (4.95%)
	Healthcare assistant: 30 (29.70%)
	Ward doctor: 14 (13.86%)

Max, Maximum observed value; min, Minimum observed value; N, Number of observations; SD, Standard Deviation.

Supplementary Table S3. General Hospital Psychiatric Unit characteristics over the study period (from 2007 to 2022). A total of 113 work accidents were recorded.

**By year**	**N (%) by-category** or **Mean ±SD [min, Max]**
Accidents	7.06 ± 4.10 [2, 16]
Physical aggressions	5.62 ±4.03 [1, 14]
Mean-presence	Nurse: 14.13 ±0.96 [12.66, 15.66]
	Healthcare assistant: 9.58 ±1.12[6.00, 10.83]
	Ward doctor: 2.60 ±0.70 [2.00, 4.08]
Personnel per bed (ratio)	Nurse: 0.94 ±0.06 [0.84, 1.04]
	Healthcare assistant: 0.64 ±0.07 [0.40, 0.72]
	Ward doctor: 0.17 ±0.04 [0.13, 0.27]
Worked-time (in months)	Nurse: 169.31 ±11.487 [152, 188]
	Healthcare assistant: 114.94 ±11.596 [84, 130]
	Ward doctor: 31.19 ±8.424 [24, 49]
	Nurse and healthcare assistant: 284.25 ±18.599 [242, 309]
	All social and health workers: 315.44 ±24.536 [266, 350]
Open-door, no-restraint policy (years)	Closed (before 2015): 8 (50.00%)<br>Open (from 2015): 8 (50.00%)

Max, Maximum observed value; min, Minimum observed value; N, Number of observations; SD, Standard Deviation.

Supplementary Table S4. Accidents' characteristics over the study period (from 2007 to 2022). A total of 113 work accidents were recorded in total personnel of 101 healthcare professionals.

Measure	N (%) by-category or Mean ±SD [min, Max]
**Accident**	
Type	Physical aggression: 92 (81.42%)
	Accidental trauma: 10 (8.85%)
	Biological risk: 8 (7.08%)
	Other: 3 (2.65%)
Significant prognosis (>0 days)	Has: 50 (44.25%)
Duration of prognosis	Overall (days): 5.75 ±13.758 [0, 107]
	With at least one significant prognosis (days): 13.00 ±18.345 [1, 107]
**Personnel**	
Gender	Female: 59 (52.21%)
	Male: 54 (47.79%)
Age	Years: 46.43 ±9.409 [25.14, 64.26]
Age-group (4-levels)	25-35 y-o: 14 (12.39%)
	36-45 y-o: 30 (26.55%)
	46-55 y-o: 46 (40.71%)
	56-65 y-o: 23 (20.35%)
Schooling	Professional: 38 (33.63%)
	High school: 42 (37.17%)
	Degree 3-years: 15 (13.27%)
	Degree: 5 (4.42%)
	More than degree: 13 (11.50%)
Professional role	Registered nurse: 63 (55.75%)
	Psychiatric nurse: 7 (6.19%)
	Healthcare assistant: 38 (33.63%)
	Ward doctor: 5 (4.42%)

Max, Maximum observed value; min, Minimum observed value; N, Number of observations; SD, Standard Deviation.

Supplementary Figure S1. Association between total accidents and accidents due to physical aggression over the study period (from 2007 to 2022). A total of 113 work accidents were recorded in total personnel of 101 healthcare professionals.

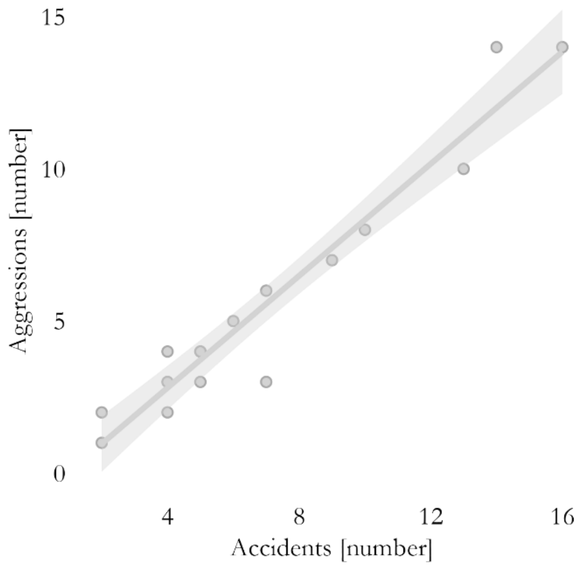

The fitting obtained with a simple regression shows mean correlation and its 95% confidence intervals (grey solid line and background).

Supplementary Figure S2. Association between accident and professionals role in healthcare professionals in the General Hospital Psychiatric Unit over the study period (from 2007 to 2022). A total of 113 work accidents were recorded in total personnel of 101 healthcare professionals.

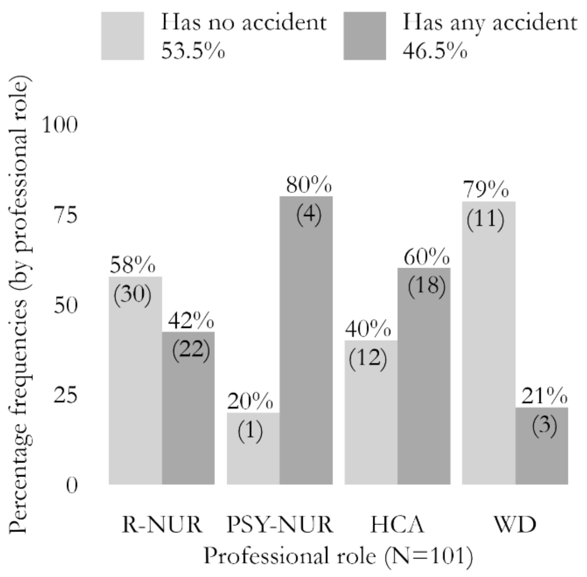

HCA, Healthcare assistant; PSY-NUR, Psychiatric nurse; R-NUR, Registered nurse; WD, Ward doctor. The number of observations is indicated in round brackets.

Supplementary Figure S3. Frequency of accidents (N=113) per year, in total personnel of 101 healthcare professionals.

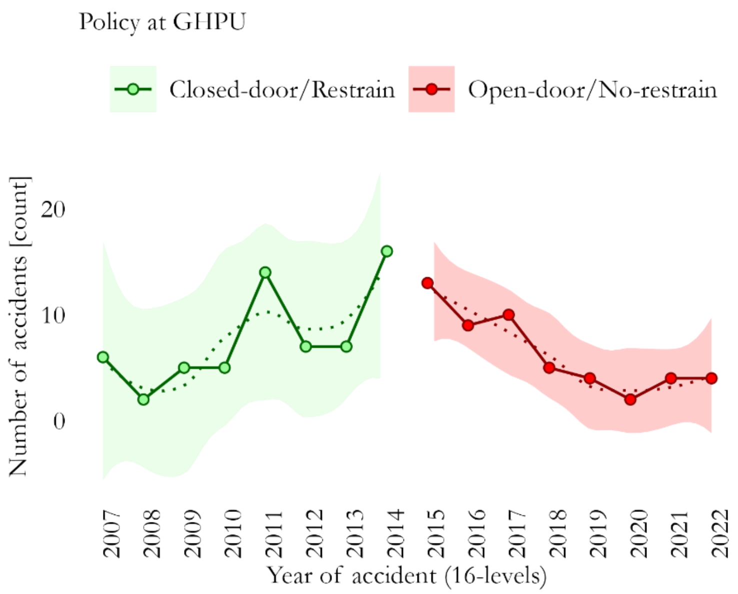

Periods with respect to the General Hospital Psychiatric Unit (GHPU) restraint policy are highlighted; the fitting obtained with a local polynomial regression shows smoothed mean frequency and its 95% confidence intervals (dotted line and background).

Supplementary Figure S4. Average number of accidents (N=113) per year according to the transition in the organizational model of the General Hospital Psychiatric Unit (GHPU) over the study period (from 2007 to 2022). Observations are organized by professional role in total personnel of 101 healthcare professionals (52 registered nurses, 63 accidents; 5 psychiatric nurses, 7 accidents; 30 healthcare assistants, 38 accidents; 14 ward doctors, 5 accidents).

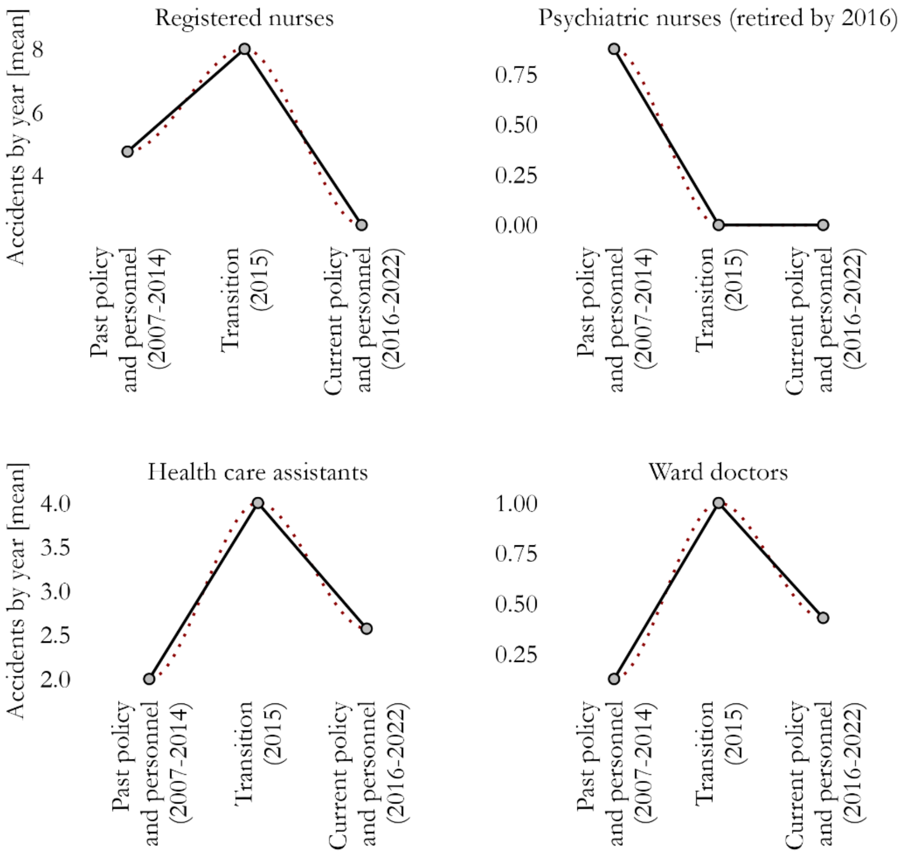

The fitting obtained with a local polynomial regression shows smoothed mean frequency (red dotted line).
